# Change in mRNA expression of sirtuin 1 and sirtuin 3 in cats fed on high fat diet

**DOI:** 10.1186/1746-6148-9-187

**Published:** 2013-09-27

**Authors:** Shingo Ishikawa, Gebin Li, Hiroshi Takemitsu, Megumi Fujiwara, Nobuko Mori, Ichiro Yamamoto, Toshiro Arai

**Affiliations:** 1 Department of Veterinary Science, School of Veterinary Medicine, Nippon Veterinary and Life Science University, 1-7-1 Kyonancho, 180-8602 Musashino, Tokyo, Japan

**Keywords:** Cat, Sirtuin, cDNA cloning, High-fat diet, Real-time PCR

## Abstract

**Background:**

Mammalian sirtuins are homologs to the yeast silent information regulator 2 (Sir2), which is an NAD-dependent deacetylase. Sirtuins are comprised of 7 proteins, and each has different target proteins. Sirtuin 1 (SIRT1) plays important roles in maintaining metabolic functions and immune responses, and SIRT3 protects cells from oxidative stress-induced cell death. Both SIRT1 and SIRT3 are regulated by metabolic status and aging. Hence, SIRT1 and SIRT3 have been researched in metabolic diseases, such as type 2 diabetes mellitus (DM), fatty liver, and heart diseases. Although these diseases have been increasing, there is little information about relation between the diseases and SIRT1 and SIRT3 in cats. Therefore we cloned SIRT1 and SIRT3 cDNA, examined mRNA expression in cat tissues, and investigated the changes in SIRT1 and SIRT3 mRNA expression in peripheral blood leukocyte of cats fed on HFD for 6 weeks.

**Results:**

Cat SIRT1 and SIRT3 contained a catalytic core region and showed high sequence homology with other vertebrate SIRT1 (>61.3%) and SIRT3 (>65.9%) amino acids. Real-time polymerase chain reaction analyses revealed that high expression levels were observed in the liver and skeletal muscle for SIRT1 and in the heart for SIRT3 in cats. In addition, both cat SIRT1 and SIRT3 expression levels in the pancreas were different between individuals. Cat SIRT1 mRNA expression in peripheral blood leukocytes was significantly elevated in obese cats fed on HFD (P < 0.05).

**Conclusions:**

Cat SIRT1 and SIRT3 genes are highly conserved among vertebrates, and HFD feeding may be related to SIRT1 mRNA expression mechanisms in cat peripheral blood leukocytes.

## Background

Mammalian sirtuins have been identified as homologs of the yeast silent information regulator 2 (Sir2) [1], which is an NAD-dependent deacetylase and related to metabolism and longevity in yeast [2]. Seven mammalian sirtuins are included in the family and each has different target proteins. Sirtuin 1 (SIRT1) is found primarily in the nucleus [3] and plays important roles in maintaining metabolic functions and immune responses through deacetylation of many substrates, such as forkhead transcription factors [4], peroxisome proliferator-activated receptor-gamma, coactivator 1-alpha [5] and the p65 subunit of nuclear factor-kappa B (NF-κB) [6]. SIRT3 is localized to mitochondria [7] and protects cells from oxidative stress-induced cell death by deacetylating isocitrate dehydrogenase 2 [8] and superoxide dismutase 2 [9]. Gene expression and activity of sirtuins are mainly regulated by metabolic states and aging [10]. These features of SIRT1 and SIRT3 have been studied in metabolic diseases related to aging such as type 2 diabetes mellitus (DM) [11,12], fatty liver [13,14], and heart diseases [15,16].

Prevalence of obesity has increased in cats, and obesity leads to the development of DM [17] and hepatic lipidosis [18]. Because the clinical, physiological, and pathological features of DM in cats closely resemble those in humans, cats are good animal models for human type 2 DM [19]. However, very little information is available on cat SIRT1 and SIRT3. The aims of this study were to determine the cDNA sequences, and examine the SIRT1 and SIRT3 mRNA expression in several tissues (Experiment 1), and to investigate the effects of feeding a high-fat diet (HFD) on the SIRT1 and SIRT3 expression (Experiment 2) in cats.

## Results

### Experiment 1

#### ***CDNA cloning of cat SIRT1 and SIRT3***

**Figure 1:**
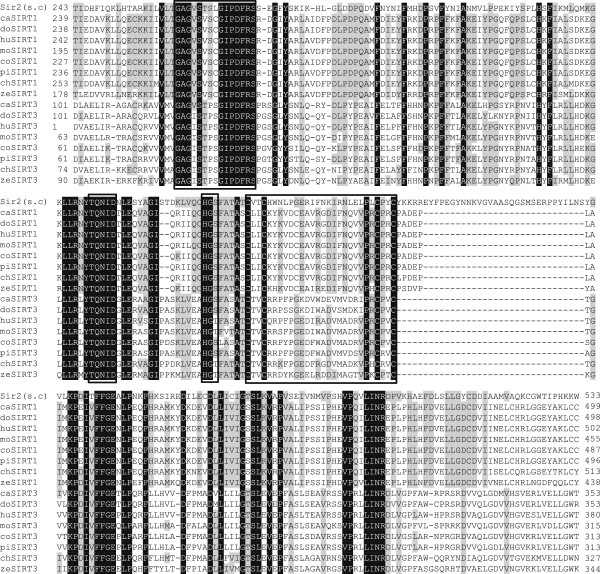
**Multiple alignment of the deduced amino acid sequences of silent information regulator 2 (Sir2) like family core region.** The deduced amino acid sequences of *Saccharomyces cerevisiae* (s.c) Sir2 (NP_010242.1), cat (c) sirtuin (SIRT)1 and 3, dog (do) SIRT1 (XP_546130.2) and 3 (XP_855809.1), human (hu) SIRT1 (NP_036370.2) and 3 (NP_036371.1), mouse (mo) SIRT1 (NP_001153061.1) and 3 (NP_001171275.1), cow (co) SIRT1 (NP_001179909.1) and 3 (NP_001193598.1), pig (pi) SIRT1 (NP_001139222.1) and 3 (NP_001103527.1), chicken (ch) SIRT1 (NP_001004767.1) and 3, (NP_001186422.1), and zebrafish SIRT1 (XP_001334440.4) and 3 (NP_001073643.1). Starting and ending residue numbers are shown. Black shaded background indicates 100% homology, whereas gray shaded backgrounds indicate >50% homology. The GAGxSxxxGIPDFR, TQNID, HG(S/T), and CxxC-(18–20)x-CxxC motifs are indicated with the box. The nucleotide sequences appear in the GenBank database with accession numbers (caSIRT1) and (caSIRT3).

**Figure 2:**
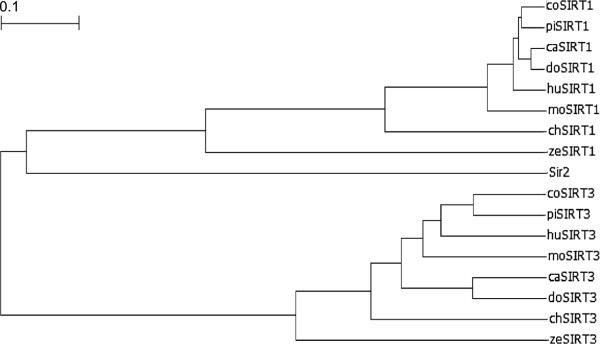
**Phylogenetic tree of the silent information regulator 2 (Sir2) like protein family.** The evolutionary tree of cow (co), pig (pi), dog (do), cat (ca), human (hu), mouse (mo), chicken (ch), zebrafish (ze), and *Saccharomyces cerevisiae* (s.c) sequences for sirtuin (SIRT)1, SIRT3, and Sir2 was made with the unweighted pair group method with arithmetic mean method using GENETYX-win Ver.9.1.0 (GENETYX Corp). The data base accession nos. for each amino acid sequence used in this analysis are described in Figure 1. The bars and values of 0.1 in the figure represent evolutionary distances.

Cat SIRT1 and SIRT3 were cloned from a cat cerebral cortex cDNA library. The cat SIRT1 cDNA consisted of a 63 bp 5′-untranslated region (UTR), a 2241 bp open reading frame (ORF), which encoded a 746 amino acids, and a 1781 bp 3′-UTR. The calculated molecular mass of this protein was 81.8 kDa. The cat SIRT3 cDNA sequence consisted of a 54 bp 5′-UTR, a 1119 bp ORF, which encoded 372 amino acids, and a 481 bp 3′-UTR. The calculated molecular mass of this protein was 40.9 kDa. Both cat SIRT1 and SIRT3 had a potential polyadenylation signal near the 3′-end (data not shown). Sequence alignment of the deduced cat SIRT1 and SIRT3 amino acids indicated that they contained a conserved catalytic core region and exhibited high homology with the corresponding region in Sir2 like proteins (Figure 1). In addition, similar to others, the cat SIRT1 and SIRT3 core region had a zinc finger and NAD^+^ binding sites. The deduced cat SIRT1 and SIRT3 amino acids sequences were compared with those of other vertebrates, which revealed high sequence similarity (SIRT1: 95.3% [with dog], 88.0% [with human], 83.2% [with mouse], 91.3% [with cow], 91.4% [with pig], 67.4% [with chicken], and 61.3% [with zebrafish]; SIRT3: 83.0% [with dog], 76.6% [with human], 73.7% [with mouse], 68.9% [with cow], 78.3% [with pig], 66.0% [with chicken], and 65.9% [with zebrafish]). In the phylogenic analysis, the evolutionary positions of cat SIRT1 and SIRT3 were located at the mammalian SIRT1 and SIRT3 branches, respectively (Figure 2).

#### ***Cat SIRT1 and SIRT3 mRNA expression profiles in tissues***

**Figure 3:**
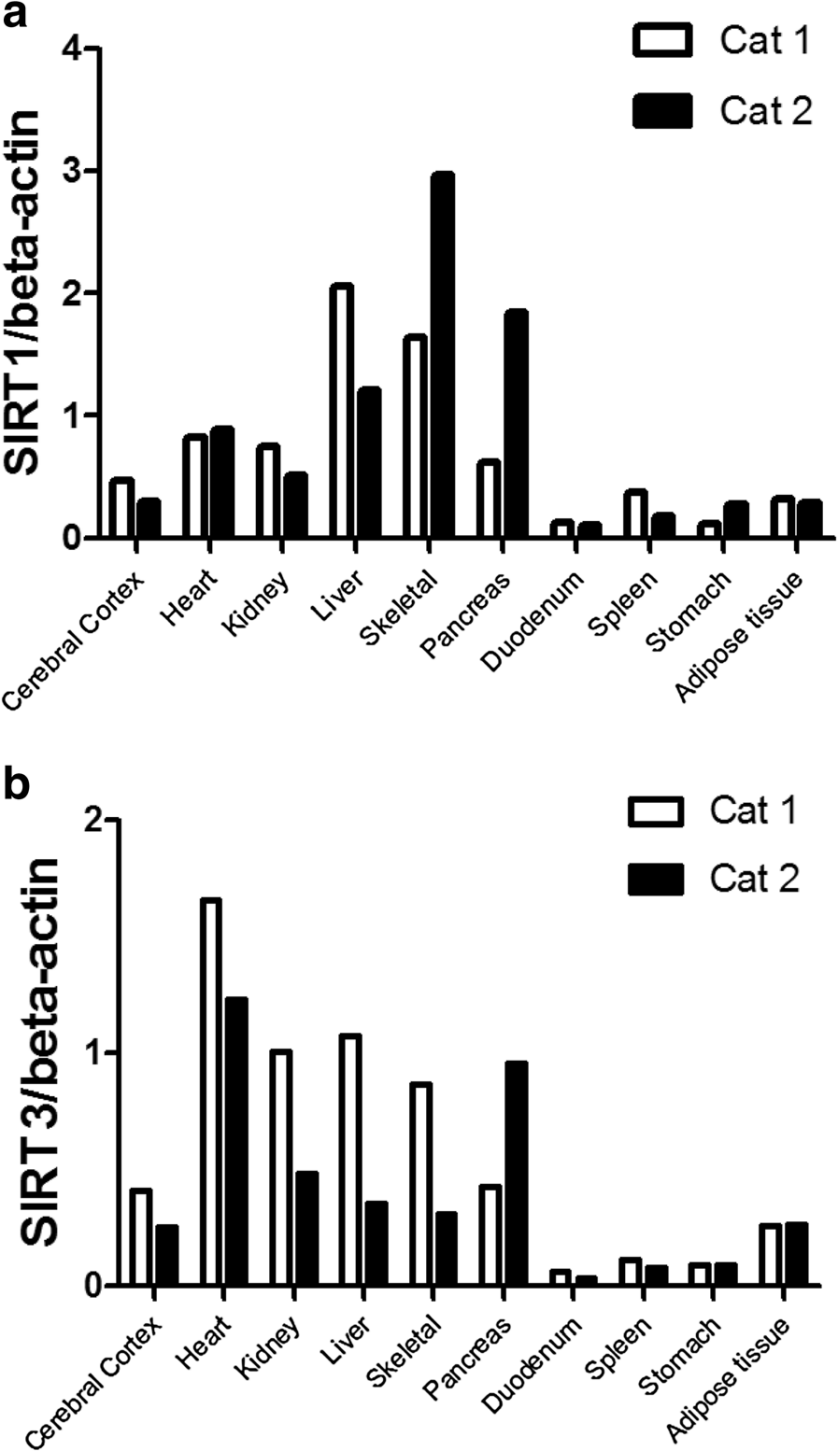
**Tissue distribution profile of cat sirtuin (SIRT)1 and SIRT3 mRNA.** Expression levels of **(a)** SIRT1 and **(b)** SIRT3 in tissues of two 3- year-old male cat (cat 1; white box bars, cat 2; black box bars) were determined by quantitative polymerase chain reaction. Each SIRT1 and SIRT3 value was normalized to that of beta-actin mRNA.

SIRT1 and SIRT3 mRNA expression levels in cat tissues were examined by quantitative real time PCR (q-PCR) (Figure 3). In two 3-year-old male cats, expression of both mRNAs were observed in a wide range of tissues, including the cerebral cortex, heart, kidneys, liver, skeletal muscles, pancreas, duodenum, spleen, stomach and adipose tissue. High expression levels were observed in the liver and skeletal muscle for SIRT1 and in the heart for SIRT3 in cats. In addition, both cat SIRT1 and SIRT3 expression levels in the pancreas were different between individuals.

### Experiment 2

#### ***Changes in cat SIRT1 and SIRT3 gene expression after HFD feeding***

**Figure 4:**
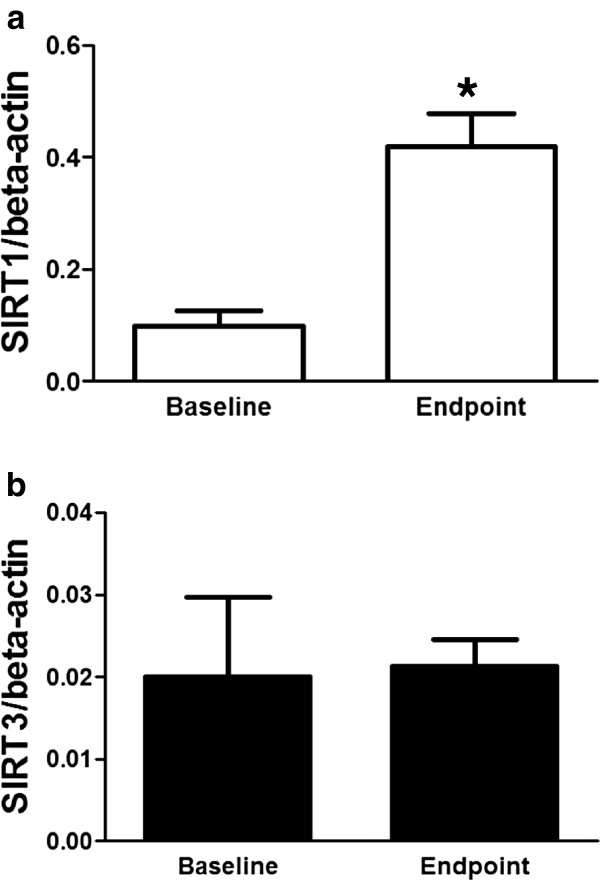
**Effect of a high-fat diet on mRNA levels of cat sirtuin (SIRT)1 and SIRT3.** Prior to the 8-week feeding period (Baseline) and the conclusion of the 8-week feeding schedule (Endpoint), SIRT1 (**a**; white box bars) and SIRT3 (**b**; black box bars) mRNA expression levels in peripheral blood leucocytes were determined by the quantitative polymerase chain reaction assay. Each SIRT1 and SIRT3 value was normalized to that of beta-actin mRNA and the mean ± standard error of mean (n = 5) for an individual RNA sample. Statistical analysis was performed using Student’s paired t-test; *P < 0.05.

We fed HFD to healthy cats for 6 weeks to examine the effect of HFD on cat SIRT1 and SIRT3 gene expression. Clinical characteristics and plasma metabolite concentrations are provided in Table 1. HFD caused significant increases in BW and hepatocellular injury markers (ALT, AST, and ALP) compared with those at baseline (P < 0.01). Peripheral blood leukocyte SIRT1 mRNA expression levels in cats significantly increased (P < 0.05) compared with those at baseline (Figure 4a). However, SIRT3 expression was not significantly different between the two conditions.

**Table 1:** Clinical characteristics and plasma metabolite concentrations

	**Baseline**	**Endopint (wk6)**
Body weight (kg)	2.6 ± 0.2	3.2 ± 0.3**
Total cholesterol (mg/dL)	100.6 ± 4.3	100.6 ± 9.4
Alanine aminotransferase (U/L)	41.6 ± 4.7	69.6 ± 6.8**
Alkaline phosphatase (U/L)	76.6 ± 14.5	101.2 ± 12.2**
Aspartate aminotransferase (U/L)	24.8 ± 1.3	32.2 ± 1.1**
Lactate dehydrogenase (U/L)	141.8 ± 21.9	131.4 ± 19.9
Serum total protein (g/dL)	6.5 ± 0.2	6.7 ± 0.2
Glucose (mg/dL)	72.6 ± 3.1	77.3 ± 0.6
Blood urea nitrogen (mg/dL)	20.0 ± 1.5	21.4 ± 0.9
Creatinine (mg/dl)	0.9 ± 0.2	1.0 ± 0.1

Values are presented as mean ± SEM.

**P < 0.01.

## Discussion

We successfully cloned the cat SIRT1 and SIRT3 cDNAs. Sequence alignment of the cat SIRT1 and SIRT3 amino acids revealed that they contained a conserved catalytic core domain [20]. This core domain included the motifs CxxC-(18–20)x-CxxC, which are known to be involved in zinc fingers, and conserved in all Sir2-like enzymes [21]. The other highly conserved motifs GAG(I/V)SxxxG(I/V)PDFRS, TQNID, and HG(S/T) create NAD^+^ binding sites [22]. SIRT1 and SIRT3 were genetically conserved in the phylogenetic tree, and may have an enzymatic function in cats.

SIRT1 and SIRT3 mRNA are expressed in a variety of tissues in humans [1,7], mice [23], cows, [24] and pigs [25]. In our study, cat SIRT1 and SIRT3 mRNA was expressed in various tissues similar to other animals, and high expression levels were observed in the liver and skeletal muscle for SIRT1 and in the heart for SIRT3 in cats. In an SIRT1 heterozygous knockout mice study, acceleration of hepatic steatosis and increased inflammatory gene expressions were observed in the liver [26]. Liver expression of cat SIRT1 may be related to control fatty acid homeostasis. Since SIRT1 enhance skeletal muscle insulin sensitivity in mice [27], a high SIRT1 mRNA expression levels in the skeletal muscle is considered to be related to glucose metabolism in cats. Although SIRT3 deficient mice appear to have normal activity, they show signs of cardiac hypertrophy at 8 weeks of age [28]. Cat SIRT3 expression in the heart may be related to protection against cardiac hypertrophy. Hypertrophic cardiomyopathy (HCM) is the most common heart disease in cats and remains a major cause of morbidity and mortality associated with the risk of sudden death [29]. Therefore, we think it might be interesting to study the relationship between SIRT3 and HCM in cats. Additionally, both SIRT1 and SIRT3 expression levels in the pancreas were different between individuals. SIRT1 regulate insulin secretion in pancreatic β cells [30] and SIRT3 is suppressed in pancreatic islets isolated from human type 2 diabetic patients [31]. Therefore, the expression levels of SIRT1 and SIRT3 in the pancreas may be considered to be fluctuating by the metabolic state in cats.

Sirtuins are regulated by nutritional status, for instance, caloric restriction up-regulates SIRT1 [32] and SIRT3 [8] activity in cultured mammalian cells. In contrast, obesity and HFD reduce SIRT1 [13] and SIRT3 [14] activity in vivo. SIRT1 protein expression is down-regulated in the liver of rat and adipose tissue of mice [13,33], whereas expression of cat SIRT1 mRNA was up-regulated in peripheral blood leukocytes by HFD. These differences in expression patterns may be considered to be differences in reactivity to HFD between tissues. In our study, early phase liver inflammation was inferred because hepatocellular injury markers were up-regulated by HFD. Some inflammatory factors are released in greater amounts from adipose tissue in obese subjects and cause chronic inflammation in animals [34,35]. HFD triggers a pro-inflammatory effect, and induces SIRT1 cleavage in adipose tissue [33]. On the other hand, pro-inflammatory factors enhance the NF-κB signal, and SIRT1 mRNA is also up-regulated as part of a feedback mechanism [6]. In addition, SIRT1 inhibits inflammatory pathways in macrophages to regulate inflammatory responses [36]. Hence we propose the hypothesis that HFD induces early phase inflammation in tissues such as liver and adipose tissue, and cat SIRT1 mRNA expression levels is up-regulated in peripheral blood leukocytes to suppress inflammation. Since peripheral blood leukocytes SIRT1 mRNA level was increase before total cholesterol and glucose level is increase, SIRT1 may become a candidate marker for early diagnosis of metabolic diseases including obesity in cats.

## Conclusions

Our study reveals the full length cat SIRT1 and SIRT3 by cDNA cloning and found that these SIRTs were highly conserved among vertebrates. And the mRNA expression analysis revealed that high expression levels were observed in the liver and skeletal muscle for SIRT1 and in the heart for SIRT3 in cats. In addition, both cat SIRT1 and SIRT3 expression levels were different between individuals. Our results provide fundamental information to reveal the cat SIRT1 and SIRT3 function about relationship of metabolic diseases. Furthermore, HFD affected cat SIRT1 mRNA expression in peripheral blood leukocytes. This represent HFD feeding may be related to SIRT1 mRNA expression mechanisms in cat peripheral blood leukocytes.

## Methods

### Experiment 1

#### ***Cloning of cat SIRT1 and SIRT3 cDNA***

Total RNA from tissues of a cat (3-year-old male) was purchased from Zyagen (San Diego, California). The amount of RNA was measured by a spectrophotometer at 260 nm. A cDNA library was prepared from cerebral cortex RNA using the SMARTer RACE cDNA Amplification Kit (Clontech, Mountain View, CA). We referred to the human SIRT1 (GenBank accession number NM_012238) and SIRT3 (GenBank accession number NM_012239) cDNA sequences and the cat genome DNA sequence to design specific primers for cat SIRT1 and SIRT3. We designed primers 1 and 2 for SIRT1 and 7 and 8 for SIRT3 to obtain the partial cat cDNA sequence (Table 2). Primers 3 and 9 were to amplify the 3′ ends of the cat SIRT1 and SIRT3 cDNA sequences respectively, and primers 4 and 10 were used for 5′ rapid amplification of cDNA ends polymerase chain reaction (RACE-PCR). Thirty cycles of PCR were performed at 98°C for 10 s, 60°C for 15 s, and 68°C for 1 min/kb with PrimeSTAR GXL DNA polymerase (Takara, Shiga, Japan), and 0.2 μM of each of the primers. The amplified fragment was cloned into the pCR-Blunt II-TOPO vector (Invitrogen, Carlsbad, CA) and the cDNA sequence was determined by a commercial DNA sequencing service (FASMAC Co., Ltd., Kanagawa, Japan).

**Table 2:** Sequences and kind of primers used for polymerase chain reaction

**Primer**	**Kind**	**Sequence (5′-3′)**	**Applications**	**Position**
SIRT1				
1	Sense	GAGAGGCAGTTGGAAGATGG	RT-PCR	47
2	Antisense	CTGTTGCTTCCTGTTTCACG	RT-PCR	2275
3	Sense	CAACGGTTTGGAAGACGATGCTG	3′ RACE	2156
4	Antisense	TCTTCCTCCTCTTCGCCCTCGTCGT	5′ RACE	452
5	Sense	CGCCTTGCAATAGACTTCCC	q-PCR	897
6	Antisense	TGAATTTGTGACAGAGAGATGGTTG	q-PCR	1042
SIRT3				
7	Sense	AGGACCTAGCTGAGCTGATTCG	RT-PCR	349
8	Antisense	TGTGTGTAGAGCCGCAGAAG	RT-PCR	656
9	Sense	CTATTTCCTCCGCCTGCTCCACGAC	3′ RACE	603
10	Antisense	AGGCCGCTCCTTGGAGACCTGAAGT	5′ RACE	464
11	Sense	TGCTTCTGCGGCTCTACAC	q-PCR	635
12	Antisense	TGTCTCCCCAAAGAACACGA	q-PCR	864
Beta-actin				
13	Sense	GCCAACCGTGAGAAGATGACT	q-PCR	353
14	Antisense	CCCAGAGTCCATGACAATACCAG	q-PCR	481

#### ***Quantitative real-time PCR (q-PCR) analysis of SIRT1 and SIRT3 in various cat tissues***

Total RNA (1 μg) was reverse transcribed by QuantiTect Reverse Transcription Kit (Qiagen, Hilden, Germany). Genomic DNA was removed by DNase treatment, and cDNA was synthesized. After inactivating the reverse transcription reaction by heating at 95°C for 3 min, the cDNA product was used for q-PCR. Reactions were carried out with Perfect Real Time SYBR Premix Ex Taq II (Takara) using an ABI 7300 Real Time PCR Sequence Detection System (Applied Biosystems, Foster City, CA) and the following Shuttle PCR protocol: 95°C for 30 s, followed by 40 cycles of 95°C for 5 s, and 60°C for 35 s, in 20 μl reaction volumes containing 2 μl template cDNA, 0.8 μl primers (0.4 μl of each), 10 μl of SYBR Premix Ex Taq II, 0.4 μl ROX Reference Dye, and 6.0 μl distilled water. Primers 5 and 6 and 11 and 12 were designed from the cloned SIRT1 and SIRT3 sequences respectively. Primers 13 and 14 were used for beta-actin mRNA. Following the real-time PCR, the fragment was subjected to dissociation-curve analysis to avoid nonspecific PCR amplification. Quantitative measurements were performed by establishing a linear amplification curve from serial dilutions of the plasmid containing cat SIRT1, SIRT3, and beta-actin cDNA fragments.

### Experiment 2

#### ***Animals***

This experiment was conducted with 5 domestic female cats (mean age, 14.0 ± 1.4 months; age range, 10–30 months; body weight [BW], 2.5 ± 0.1 kg). Veterinarians confirmed that the cats were healthy and without any clinical manifestations. All cats were individually housed and maintained for 6 weeks at AQS Co. Ltd. (Narita, Japan). The cats were fed on HFD, which was made to order by Nippon Pet Food, Inc. (Tokyo, Japan). The composition of HFD was moisture (7.0%), crude protein (32.7%), crude fat (23.9%), crude fiber (0.9%), crude ash (5.5%), and nitrogen free extract (29.9%). The caloric content was 4660 kcal/kg. The fatty acid composition of HFD was 14:0 (1.3%), 14:1 (0.4%), 15:0 (0.2%), 16:0 (22.6%), 16:1 (2.3%), 17:0 (0.4%), 17:1 (0.3%), 18:0 (23.1%), 18:1 (35.7%), 18:2n-6 (10.9%), 18:3n-3 (0.4%), 20:0 (0.5%), 20:1 (0.3%), 20:4n-6 (0.2%), 22:0 (0.2%), and unidentified (1.2%). The cats were fed the diet *ad libitum* for their daily energy requirement (DER) from 9:00 AM to 8:30 AM the next day. Any surplus diet was removed at 4:00 PM a day prior to blood sampling. DER was calculated as 1.4 × resting energy requirement (RER) (BW^0.75^ × 70). RER was based on BW before the meal at 9:00 AM. Cats were housed in individual cages and provided with water *ad libitum*. The animal room was maintained at 24 ± 2°C and 55 ± 10% relative humidity on a 12:12 h light: dark cycle (lights on from 8:00 AM to 8:00 PM). Approval for this study was provided by the Nippon Veterinary and Life Science University Animal Research Committee.

### Blood sampling

Pre-prandial blood samples (4–5 ml) were withdrawn from the jugular vein of cats without sedation prior to the 6 week feeding period (Baseline); 2.5 ml of this blood was collected in PAX gene RNA tubes (PreAnalytiX, Hombrechtikon, Switzerland) for RNA stabilization, preservation, and sample transport, and the remainder was collected in heparinized tubes, for immediate centrifugation at 1500 × *g* for 10 min at 4°C to obtain plasma, which was stored at -30°C until analysis. At the conclusion of the 6 week feeding schedule (Endpoint), pre-prandial blood (5 ml) was withdrawn again from the same site and treated in the same manner.

### Plasma metabolite and hepatic injury marker enzyme analysis

Plasma total cholesterol, total protein, glucose, blood urea nitrogen, and creatinine concentrations as well as alanine aminotransferase (ALT), alkaline phosphatase (ALP), asparate aminotransferase (AST), and lactate dehydrogenase activities were determined using an autoanalyzer (AU680, Beckman Coulter, CA, USA).

### q-PCR analysis of peripheral blood leukocyte mRNA

Total leukocyte RNA was extracted from the blood samples using TRIzol (Invitrogen), according to the manufacturer’s protocol. RNA concentration was assessed by spectrophotometer at 260 nm, and the presence of isolated RNA was assessed by native agarose gel electrophoresis on a 0.8% agarose gel. The cDNA synthesis and q-PCR analysis were performed as described above.

### Statistical analysis

Data are presented as mean ± standard error of mean (SEM) and were analyzed using Student’s paired t-test. All analyses were performed using GraphPad Prism (GraphPad Software, San Diego, CA). A *P* < 0.05 was considered significant.

## Competing interests

None of the authors has any financial or personal relationships that could inappropriately influence or bias the content of the paper.

## Authors’ contributions

SI designed the study, performed experiments, analyzed data, and drafted the manuscript. GL, MF, HT, and NM participated in data collection and experimental procedure. IY helped with study design and data analysis. TA contributed to the study design and helped with editing and revision of the manuscript. All authors read and approved the final manuscript.

## References

[B1] Frye RA Characterization of five human cDNAs with homology to the yeast SIR2 gene: Sir2-like proteins (sirtuins) metabolize NAD and may have protein ADP-ribosyltransferase activity Biochem Biophys Res Commun 1999 260 1 273 279 10.1006/bbrc.1999.0897 10381378

[B2] Howitz KT Bitterman KJ Cohen HY Lamming DW Lavu S Wood JG Zipkin RE Chung P Kisielewski A Zhang LL Scherer B Sinclair DA Small molecule activators of sirtuins extend Saccharomyces cerevisiae lifespan Nature 2003 425 6954 191 196 10.1038/nature01960 12939617

[B3] Michishita E Park JY Burneskis JM Barrett JC Horikawa I Evolutionarily conserved and nonconserved cellular localizations and functions of human SIRT proteins Mol Biol Cell 2005 16 10 4623 4635 10.1091/mbc.E05-01-0033 16079181 PMC1237069

[B4] Brunet A Sweeney LB Sturgill JF Chua KF Greer PL Lin Y Tran H Ross SE Mostoslavsky R Cohen HY Hu LS Cheng HL Jedrychowski MP Gygi SP Sinclair DA Alt FW Greenberg ME Stress-dependent regulation of FOXO transcription factors by the SIRT1 deacetylase Science 2004 303 5666 2011 2015 10.1126/science.1094637 14976264

[B5] Rodgers JT Lerin C Haas W Gygi SP Spiegelman BM Puigserver P Nutrient control of glucose homeostasis through a complex of PGC-1alpha and SIRT1 Nature 2005 434 7029 113 118 10.1038/nature03354 15744310

[B6] Zhang HN Li L Gao P Chen HZ Zhang R Wei YS Liu DP Liang CC Involvement of the p65/RelA subunit of NF-kappaB in TNF-alpha-induced SIRT1 expression in vascular smooth muscle cells Biochem Biophys Res Commun 2010 397 3 569 575 10.1016/j.bbrc.2010.05.160 20617556

[B7] Onyango P Celic I McCaffery JM Boeke JD Feinberg AP SIRT3, A human SIR2 homologue, is an NAD-dependent deacetylase localized to mitochondria Proc Natl Acad Sci U S A 2002 99 21 13653 13658 10.1073/pnas.222538099 12374852 PMC129731

[B8] Someya S Yu W Hallows WC Xu J Vann JM Leeuwenburgh C Tanokura M Denu JM Prolla TA Sirt3 Mediates reduction of oxidative damage and prevention of age-related hearing loss under caloric restriction Cell 2010 143 5 802 812 10.1016/j.cell.2010.10.002 21094524 PMC3018849

[B9] Qiu X Brown K Hirschey MD Verdin E Chen D Calorie restriction reduces oxidative stress by SIRT3-mediated SOD2 activation Cell Metab 2010 12 6 662 667 10.1016/j.cmet.2010.11.015 21109198

[B10] Longo VD Kennedy BK Sirtuins in aging and age-related disease Cell 2006 126 2 257 268 10.1016/j.cell.2006.07.002 16873059

[B11] Banks AS Kon N Knight C Matsumoto M Gutiérrez-Juárez R Rossetti L Gu W Accili D SirT1 Gain of function increases energy efficiency and prevents diabetes in mice Cell Metab 2008 8 4 333 341 10.1016/j.cmet.2008.08.014 18840364 PMC3222897

[B12] Jing E Emanuelli B Hirschey MD Boucher J Lee KY Lombard D Verdin EM Kahn CR Sirtuin-3 (Sirt3) regulates skeletal muscle metabolism and insulin signaling via altered mitochondrial oxidation and reactive oxygen species production Proc Natl Acad Sci U S A 2011 108 35 14608 14613 10.1073/pnas.1111308108 21873205 PMC3167496

[B13] Deng XQ Chen LL Li NX The expression of SIRT1 in nonalcoholic fatty liver disease induced by high-fat diet in rats Liver Int 2007 27 5 708 715 10.1111/j.1478-3231.2007.01497.x 17498258

[B14] Kendrick AA Choudhury M Rahman SM McCurdy CE Friederich M Van Hove JL Watson PA Birdsey N Bao J Gius D Sack MN Jing E Kahn CR Friedman JE Jonscher KR Fatty liver is associated with reduced SIRT3 activity and mitochondrial protein hyperacetylation Biochem J 2011 433 3 505 514 10.1042/BJ20100791 21044047 PMC3398511

[B15] Tanno M Kuno A Yano T Miura T Hisahara S Ishikawa S Shimamoto K Horio Y Induction of manganese superoxide dismutase by nuclear translocation and activation of SIRT1 promotes cell survival in chronic heart failure J Biol Chem 2010 285 11 8375 8382 10.1074/jbc.M109.090266 20089851 PMC2832987

[B16] Pillai VB Sundaresan NR Kim G Gupta M Rajamohan SB Pillai JB Samant S Ravindra PV Isbatan A Gupta MP Exogenous NAD blocks cardiac hypertrophic response via activation of the SIRT3-LKB1-AMP-activated kinase pathway J Biol Chem 2010 285 5 3133 3144 10.1074/jbc.M109.077271 19940131 PMC2823454

[B17] O’Brien TD Pathogenesis of feline diabetes mellitus Mol Cell Endocrinol 2002 197 1–2 213 219 12431815 10.1016/s0303-7207(02)00265-4

[B18] Armstrong PJ Blanchard G Hepatic lipidosis in cats Vet Clin North Am Small Anim Pract 2009 39 3 599 616 10.1016/j.cvsm.2009.03.003 19524794

[B19] Henson MS O’Brien TD Feline models of type 2 diabetes mellitus ILAR J 2006 47 3 234 242 10.1093/ilar.47.3.234 16804198

[B20] Sanders BD Jackson B Marmorstein R Structural basis for sirtuin function: what we know and what we don’t Biochim Biophys Acta 2010 1804 8 1604 1616 10.1016/j.bbapap.2009.09.009 19766737 PMC2886166

[B21] Sherman JM Stone EM Freeman-Cook LL Brachmann CB Boeke JD Pillus L The conserved core of a human SIR2 homologue functions in yeast silencing Mol Biol Cell 1999 10 9 3045 3059 10.1091/mbc.10.9.3045 10473645 PMC25551

[B22] Min J Landry J Sternglanz R Xu RM Crystal structure of a SIR2 homolog-NAD complex Cell 2001 105 2 269 279 10.1016/S0092-8674(01)00317-8 11336676

[B23] Shi T Wang F Stieren E Tong Q SIRT3, A mitochondrial sirtuin deacetylase, regulates mitochondrial function and thermogenesis in brown adipocytes J Biol Chem 2005 280 14 13560 13567 10.1074/jbc.M414670200 15653680

[B24] Ghinis-Hozumi Y González-Gallardo A González-Dávalos L Antaramian A Villarroya F Shimada A Varela-Echavarría A Mora O Bovine sirtuins: initial characterization and expression of sirtuins 1 and 3 in liver, muscle, and adipose tissue J Anim Sci 2011 89 8 2529 2536 10.2527/jas.2010-3476 21421831

[B25] Jin D Tan HJ Lei T Gan L Chen XD Long QQ Feng B Yang ZQ Molecular cloning and characterization of porcine sirtuin genes Comp Biochem Physiol B Biochem Mol Biol 2009 153 4 348 358 10.1016/j.cbpb.2009.04.004 19389481

[B26] Xu F Gao Z Zhang J Rivera CA Yin J Weng J Ye J Lack of SIRT1 (mammalian sirtuin 1) activity leads to liver steatosis in the SIRT1+/- mice: a role of lipid mobilization and inflammation Endocrinology 2010 151 6 2504 2514 10.1210/en.2009-1013 20339025 PMC2875813

[B27] Schenk S McCurdy CE Philp A Chen MZ Holliday MJ Bandyopadhyay GK Osborn O Baar K Olefsky JM Sirt1 Enhances skeletal muscle insulin sensitivity in mice during caloric restriction J Clin Invest 2011 121 11 4281 4288 10.1172/JCI58554 21985785 PMC3204844

[B28] Sundaresan NR Gupta M Kim G Rajamohan SB Isbatan A Gupta MP Sirt3 Blocks the cardiac hypertrophic response by augmenting Foxo3a-dependent antioxidant defense mechanisms in mice J Clin Invest 2009 119 9 2758 2771 19652361 10.1172/JCI39162 PMC2735933

[B29] Abbott JA Feline hypertrophic cardiomyopathy: an update Vet Clin North Am Small Anim Pract 2010 40 4 685 700 10.1016/j.cvsm.2010.04.004 20610019

[B30] Bordone L Motta MC Picard F Robinson A Jhala US Apfeld J McDonagh T Lemieux M McBurney M Szilvasi A Easlon EJ Lin SJ Guarente L Sirt1 Regulates insulin secretion by repressing UCP2 in pancreatic beta cells PLoS Biol 2006 4 2 e31 10.1371/journal.pbio.0040031 16366736 PMC1318478

[B31] Caton PW Richardson SJ Kieswich J Bugliani M Holland ML Marchetti P Morgan NG Yaqoob MM Holness MJ Sugden MC Sirtuin 3 regulates mouse pancreatic beta cell function and is suppressed in pancreatic islets isolated from human type 2 diabetic patients Diabetologia 2013 56 5 1068 1077 10.1007/s00125-013-2851-y 23397292

[B32] Cohen HY Miller C Bitterman KJ Wall NR Hekking B Kessler B Howitz KT Gorospe M de Cabo R Sinclair DA Calorie restriction promotes mammalian cell survival by inducing the SIRT1 deacetylase Science 2004 305 5682 390 392 10.1126/science.1099196 15205477

[B33] Chalkiadaki A Guarente L High-fat diet triggers inflammation-induced cleavage of SIRT1 in adipose tissue to promote metabolic dysfunction Cell Metab 2012 16 2 180 188 10.1016/j.cmet.2012.07.003 22883230 PMC3539750

[B34] Hotamisligil GS Inflammation and metabolic disorders Nature 2006 444 7121 860 867 10.1038/nature05485 17167474

[B35] Laflamme DP Companion animals symposium: obesity in dogs and cats: what is wrong with being fat? J Anim Sci 2012 90 5 1653 1662 10.2527/jas.2011-4571 21984724

[B36] Yoshizaki T Schenk S Imamura T Babendure JL Sonoda N Bae EJ Oh DY Lu M Milne JC Westphal C Bandyopadhyay G Olefsky JM SIRT1 Inhibits inflammatory pathways in macrophages and modulates insulin sensitivity Am J Physiol Endocrinol Metab 2010 298 3 E419 E428 10.1152/ajpendo.00417.2009 19996381 PMC2838524

